# ON-bipolar cell gene expression during retinal degeneration: Implications for optogenetic visual restoration

**DOI:** 10.1016/j.exer.2021.108553

**Published:** 2021-06

**Authors:** Michael J. Gilhooley, Doron G. Hickey, Moritz Lindner, Teele Palumaa, Steven Hughes, Stuart N. Peirson, Robert E. MacLaren, Mark W. Hankins

**Affiliations:** a Nuffield Laboratory of Ophthalmology, Nuffield Department of Clinical Neuroscience, University of Oxford, Oxford, OX1 3RE, United Kingdom; b Sleep and Circadian Neuroscience Institute, Nuffield Department of Clinical Neuroscience, University of Oxford, Oxford, OX1 3RE, United Kingdom; c Royal Victorian Eye and Ear Hospital, Melbourne, 002, Australia; d The Oxford Eye Hospital, Oxford, OX3 9DU, United Kingdom; e Moorfields Eye Hospital, London, EC1V 2PD, United Kingdom; f Institute of Physiology and Pathophysiology, Department of Neurophysiology, Philipps University, Deutschhausstrasse 1-2, Marburg, 35037, Germany

**Keywords:** Bipolar cell, Gene expression, Optogenetics, Inherited retinal degeneration

## Abstract

**Purpose:**

Retinal bipolar cells survive even in the later stages of inherited retinal degenerations (IRDs) and so are attractive targets for optogenetic approaches to vision restoration. However, it is not known to what extent the remodelling that these cells undergo during degeneration affects their function. Specifically, it is unclear if they are free from metabolic stress, receptive to adeno-associated viral vectors, suitable for opsin-based optogenetic tools and able to propagate signals by releasing neurotransmitter.

**Methods:**

Fluorescence activated cell sorting (FACS) was performed to isolate labelled bipolar cells from dissociated retinae of litter-mates with or without the IRD mutation Pde6b^rd1/rd1^ selectively expressing an enhanced yellow fluorescent protein (EYFP) as a marker in ON-bipolar cells. Subsequent mRNA extraction allowed Illumina® microarray comparison of gene expression in bipolar cells from degenerate to those of wild type retinae. Changes in four candidate genes were further investigated at the protein level using retinal immunohistochemistry over the course of degeneration.

**Results:**

A total of sixty differentially expressed transcripts reached statistical significance: these did not include any genes directly associated with native primary bipolar cell signalling, nor changes consistent with metabolic stress. Four significantly altered genes (Srm2, Slf2, Anxa7 & Cntn1), implicated in synaptic remodelling, neurotransmitter release and viral vector entry had immunohistochemical staining colocalising with ON-bipolar cell markers and varying over the course of degeneration.

**Conclusion:**

Our findings suggest relatively few gene expression changes in the context of degeneration: that despite remodelling, bipolar cells are likely to remain viable targets for optogenetic vision restoration. In addition, several genes where changes were seen could provide a basis for investigations to enhance the efficacy of optogenetic therapies.

## Highlights

• Bipolar cells are attractive targets for therapeutic optogenetics in IRDs.
• This is the first cell specific transcriptomic analysis of bipolar cells in an IRD model.
• Bipolar cells maintain expression of genes essential to act as targets for optogenetics.
• Protein staining relating to four candidate genes (Anxa7, Cntn1, Srm2, Sulf2) is confirmed using immunohistochemistry.

## Abbreviations

ANOVA: ANalysis Of VAriance
AAV: Adeno Associated Virus
EYFP: Enhanced Yellow Fluorescent Protein
FACS: Fluorescence Activated Cell Sorting
FDR: False Detection Rate
HSPG: Heparin Sulphate Proteoglycan
IHC: Immunohistochemistry
IRDs: Inherited Retinal Degenerations P40, P90, P120, P150 – Postnatal day 40, 90, 120, 150
LTD/P: Long Term Depression/Potentiation
PKC α: Protein Kinase C - α
qPCR: Quantitative Polymerase Chain Reaction

## 1 Introduction

Advances in retinal gene therapy delivery methods, such as adeno-associated virus (AAV), have allowed retinal gene replacement to become a reality for patients suffering from certain inherited retinal degenerations (IRDs) (Russell et al., 2017). With this success, attention has turned to expanding the use of these proven vectors with alternative strategies for visual restoration such as optogenetics - the expression of exogenous light sensitive proteins within an excitable cell - which may be applied to a wide range of IRDs, regardless of the causative mutation.

AAV delivered optogenetic tools have been shown to restore electrophysiological and behavioural responses to light in animal models of IRDs (Cehajic-Kapetanovic et al., 2015; De Silva et al., 2017; Doroudchi et al., 2011) by rendering surviving cells in the degenerate retina sensitive to light. This general principle of survivor cell stimulation has also been demonstrated clinically with electronic retinal prostheses already in clinical use for vision restoration (Edwards et al., 2018; Luo and da Cruz, 2016).

While effective, the stimulation of retinal ganglion cells - especially by epiretinal prostheses - bypasses much of the early image processing carried out in the retina. This makes specific stimulation of cells higher in the retinal hierarchy, such as the bipolar cell, conceptually attractive. However, significant retinal remodelling does occur after the death of the photoreceptor (Gilhooley and Acheson, 2017; Jones and Marc, 2005), potentially compromising the suitability of bipolar cells as targets for such stimulation. Indeed, changes in morphology, synaptic connections, electrophysiological responses and receptor expression (Dunn, 2015; Gayet-Primo and Puthussery, 2015; Kalloniatis et al., 2016; Marc and Jones, 2003; Marc et al., 2003, 2007; Varela et al., 2003) have been observed in human and animal studies.

To date, it is not understood how changes specifically within the bipolar cells during degeneration will affect their long-term viability as optogenetic targets. Particularly, if they are free from metabolic stress, receptive to adeno-associated viral vectors, suitable for opsin based optogenetic tools and able to propagate their signal by releasing neurotransmitter in response to exogenous optogenetic stimulation. While studies of general gene expression changes in animal models of the degenerate retina exist in the literature (Dorrell et al., 2004; Hackam et al., 2004; Hornan et al., 2007; Michalakis et al., 2013; Punzo and Cepko, 2007; Yu et al., 2018), none has considered the retinal bipolar cell in isolation.

Investigation of bipolar cells is particularly apposite for two reasons: first, the development of delivery tools to specifically target discrete retinal cell populations (such as cell specific promoters and AAV capsid tropism (Cronin et al., 2014; de Leeuw et al., 2014; de Silva et al., 2015; Juttner et al., 2019; Kleine Holthaus et al., 2020; Lu et al., 2016; Scalabrino et al., 2015)) have made cell-specific delivery a realistic possibility. Secondly, human opsins such as rhodopsin (Cehajic-Kapetanovic et al., 2015; Gaub et al., 2015), cone opsin (Berry et al., 2019), melanopsin (De Silva et al., 2017; Lin et al., 2008) and variants (van Wyk et al., 2015) are being described as sensitive optogenetic tools. These are known to couple to endogenous G protein signalling cascades (Hughes et al., 2016) allowing greater signal amplification compared to microbial opsins, such as channelrhodopsin (Lagali et al., 2008), which lack such coupling. However, this coupling could be affected by changes in levels of constituents of these cascades in bipolar cells during retinal degeneration. Therefore, investigation of retinal bipolar cells specifically in IRD models is paramount in determining if this conceptually attractive strategy of bipolar specific targeting is likely to be feasible for the clinical translation of optogenetics.

The principal objective of this study was to confirm the continued expression of the principal components of both the ON-bipolar light signalling and other second messenger cascades during IRDs. Here we show that, despite remodelling, bipolar cells undergo remarkably limited transcriptomic changes in response to the loss of synaptic inputs from photoreceptors, even in the late stages of the disease in an animal model.

The secondary aim of the study was to identify differentially expressed genes for further characterisation in both Pde6b^wt/wt^ and Pde6b^rd1/rd1^ retinae using immunohistochemistry. Together, these findings will be central to guiding investigations to effectively translate bipolar cell targeted optogenetic therapies into clinical use.

## 2 Methods

### 2.1 Mouse lines

All experiments involving animals were performed in accordance with the Animals for Scientific Procedures Act 1986, licence no. 30/3371 and approved by the University of Oxford animal welfare and ethical review body and the ARVO Statement for the Use of Animals in Ophthalmic and Vision Research. A transgenic mouse line (“L7.Cre.EYFP.Pde6b^x/x^”, supplementary methods) was used which:

1. Expressed Cre recombinase under the control of the ON-bipolar cell specific promoter “L7” (also known in the literature as “Pcp2”),
2. Were homozygous for “floxed” EYFP at the Rosa26 locus and therefore expressed EYFP in L7 (retinal ON-bipolar) cells.
3. Were either Pde6b^wt/wt^ or Pde6b^rd1/rd1^ (wild type or retinal degeneration phenotype)

### 2.2 Isolation of RNA from retinal bipolar cells and comparison with gene array

**Fig. 1:**
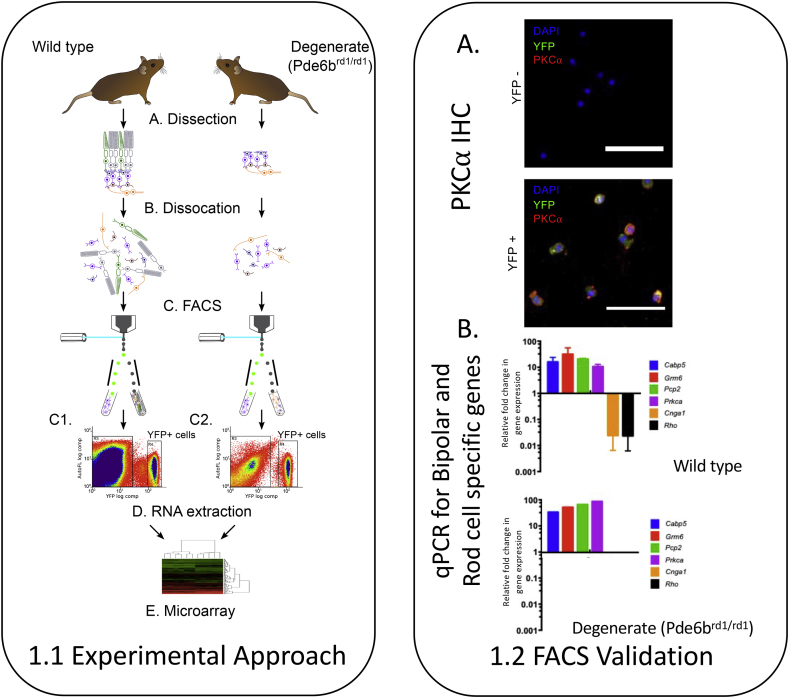
1.1 Experimental Approach: fluorescence-activated cell sorting for a microarray study of retinal bipolar cells from degenerate retinas. (A)L7-Cre EYFP non-degenerate (Pde6b^wt/wt^) and degenerate (Pde6b^rd1/rd1^) mice were culled at P90 and the retinae removed with careful dissection to remove Retinal Pigment Epithelium before dissociation (B) FACS was then used to isolate YFP-positive (ON-Bipolar) cells (C). From this isolate, RNA was extracted (D) and used for a microarray study. Panel (C1) represents an illustrative FACS dot plot of a single dissociated, non-degenerate L7.Cre.EYFP retinae and (C3) the same from a degenerate retina. 1.2 FACS Validation. Methods to confirm the identity of isolated YFP + cells. (A) Immunocytochemistry (ICC) of YFP positive and YFP negative FACS isolate stained for DAPI (blue), PKCα (red) and YFP (green) showing, as expected enrichment of PKCα + (bipolar) cells in the YFP + isolate. Scale bars = 50 μm. (B) Quantitative PCR (qPCR) comparing expression of retinal bipolar cell-specific and rod cell-specific genes in the YFP-positive cell population, relative to the YFP- negative cell population in samples from a small number of wild type and degenerate (Pde6b^rd1/rd1^) L7-Cre EYFP retinas following FACS at P90. The YFP-positive cell fraction had higher expression of bipolar-specific and lower expression of rod-specific genes, indicating that the YFP-positive population was enriched in bipolar cells (mean ± s.e.m.; n = 2, non-degenerate, n = 1, degenerate). (For interpretation of the references to colour in this figure legend, the reader is referred to the Web version of this article.)

Six L7.Cre.EYFP.Pde6b^wt/wt^ and Six L7.Cre.EYFP.Pde6b^rd1/rd1^ mice underwent cervical dislocation at P89 to P91, 6 h into their light phase with immediate enucleation. All mice were littermates, three of each group were female. Retinae were dissected with special care to remove retinal pigment epithelium before cell dissociation using a papain dissociation kit (Worthington Biochemical, Lakewood, USA) according to the manufacturer's instructions (Fig. 1).

The resulting dissociated cells were subjected to fluorescence activated cell sorting (FACS) (Fig. 1) with 97.3 ± 1.8% of cells in the YFP + isolate co-staining for PKCα on ICC. RNA was extracted from cell isolates and processed using standard methods for use on a MouseWG-6 v2 Expression BeadChip (Illumina). Expression levels were compared using Lumi (Du et al., 2008) and Limma (Ritchie et al., 2015) packages for R (Team, 2013) with quintile normalisation. Results were corrected for multiple testing using FDR testing (Benjamini, 1995) (supplementary methods).

When isolating dissociated retinal cells in such a way, the large number of rod photoreceptor cells in the wild type retina as well as their invaginated bipolar cell synapses could potentially lead to rods being carried over with labelled bipolar cells into the isolate. Increasing the length of enzymatic dissociation can minimise rod contamination, yet such approaches must be weighed against the risk of damage to the isolated cells or RNA with extended papain dissociation times. These protocols not only require longer periods for cells in a ‘non-physiological’ state before mRNA extraction, but lead to greater loss of cell processes (dendrites and axons). These are especially relevant to bipolar cells given the dramatic changes to these parts of the cell during degeneration.

Previous studies have attempted to control for rod carry over in different ways - for example, Siegert et al. (2012) normalise all gene expression levels based on expression of known rod specific genes whereas Punzo et al.(Punzo and Cepko, 2007), exclude any known rod specific genes from further analysis while Berg et al. (2019) examine changes in array expression of known photoreceptor genes to quantify levels of contamination.

We specifically interrogated our samples (prior to gene array) for two known rod specific genes (Rho & Cnga1) in a small number of samples using qPCR to confirm relative sample purity (supplementary methods, Fig. 1). Despite this, our gene array data did present differential expression of a small number of genes with either a rod ontological annotation or where previous literature reported low or absent protein expression in bipolar cells compared to photoreceptors (Table S1). To avoid difficulties in interpretation, we excluded these genes from further investigation (Berg et al., 2019; Punzo and Cepko, 2007) but did not systematically alter our data otherwise (Siegert et al., 2012). Like all of the approaches described above, this relies on an (fortunately available) a priori knowledge of gene photoreceptor specificity which should be borne in mind when extrapolating conclusions beyond this model.

### 2.3 Identifying candidate genes for further investigation

As in previous studies, we combined several practical strategies to prioritise those probes found to have significantly different expression for further investigation (Berg et al., 2019; Michalakis et al., 2013; Punzo and Cepko, 2007; Siegert et al., 2012). After removal of genes with rod annotations (Table S1) interaction & pathway prediction software (Fabregat et al., 2016; Warde-Farley et al., 2010) was used to identify shared functions or pathways linking groups of differentially expressed genes. All differentially expressed genes were also searched for on the RetNet (Daiger et al., 1998) database of human retinal disease genotype-phenotype relations to identify potentially clinically relevant candidates.

For each candidate, a search was performed on the NCBI® Gene database to extract gene ontology annotations and on the Medline® database allow systematic review of the literature (supplementary methods). Gene ontology terms were used to group the genes into six broad functional groups (Table 1) while review of primary literature allowed prioritisation of an initial candidate in each group for further investigation (supplementary methods) and identification of any potential photoreceptor specific genes (Table S1). Within each of first four groups, one candidate was prioritised (supplementary methods) to be further characterised by IHC staining.

**Table 1:** Genes differentially expressed between Pde6b^rd1/rd1^ and Pde6b^+/+^ retinae at P90, grouped by broad function based on gene ontology terms annotated to the gene's entry on the gene ontology consortium database (two listed for each gene). Δ = change in expression compared to Pde6b^wt/wt^ (i.e. ‘+’ = up regulated; ‘-’ = down regulated) please see Table S2 for corresponding FDR adjusted p-values.

Symbol	Δ log2	Ontology term 1	Ontology term 2
A - Actin cytoskeleton, microtubules and intracellular transport			

Ap3m2	−1.85	anterograde synaptic vesicle transport	intracellular protein transport
Cng	3.52	microtubule cytoskeleton organisation	microtubule binding
Sept4	1.54	cilium assembly	mitotic cytokinesis
**Srm2**	**1.81**	**actin cytoskeleton organisation**	**melanosome organisation**
Tmsb10	−2.15	actin cytoskeleton organisation	regulation of cell migration
B - Heparin sulphate proteoglycan metabolism			

Extl3	−2.05	heparan sulphate proteoglycan biosynthesis	protein glycosylation
**Slf2**	**1.43**	**heparan sulphate proteoglycan metabolic process**	**arylsulfatase activity**
C - Cell signalling, calcium homeostasis			
**Anxa7**	**−1.56**	**calcium ion binding**	**membrane fusion**
Cabyr	2.25	sperm capacitation	calcium-mediated signalling
Gabrg2	−1.56	gamma-aminobutyric acid signalling pathway	synaptic transmission, GABAergic
Pde1c	−1.96	signal transduction	response to calcium ion
Slc7a3	−2.87	amino acid transport	arginine transport
Unc13a	−2.11	neurotransmitter secretion	SNARE binding
D - Neural cell growth, survival & remodelling			
**Cntn1**	**−1.79**	**neuron projection development**	**nervous system development**
Efnb1	2.15	axon guidance	presynapse assembly
Hdac9	−3.69	DNA repair	chromatin organisation
Lynx1	−8.26	synaptic transmission, cholinergic	acetylcholine receptor binding
Mif	−1.91	regulation of cell proliferation	positive regulation of axon regeneration
Nrm	−1.40	nuclear membrane	membrane
Pcdha7	−1.98	cell adhesion	cell-cell recognition
Phc1	1.93	cellular response to retinoic acid	histone ubiquitination
Ptprr	−3.72	negative regulation of ERK1 and ERK2 cascade	ERBB2 signalling pathway
Sfrs1	1.72	mRNA 5′-splice site recognition	regulation of transcription, DNA-templated
Socs5	−1.64	regulation of growth	JAK-STAT cascade
Yaf2	−2.54	regulation of transcription, DNA-templated	transcription, DNA-templated
E - Aerobic and anaerobic respiration, cellular response to stress			

Atox1	−1.62	transition metal ion binding	response to oxidative stress
Clip1	−2.75	transition metal ion binding	microtubule bundle formation
Cox3	−1.83	aerobic electron transport chain	aerobic respiration
Msrb2	3.03	transition metal ion binding	cellular response to stress
Mt1	−10.73	transition metal ion binding	cellular metal ion homeostasis
Ndufb2	−1.55	mitochondrial respiratory chain complex I	oxidation-reduction process
Pgk1	−2.30	glycolytic process	carbohydrate metabolic process
F - Miscellaneous			

Abca1	−3.10	cholesterol homeostasis	cellular response to retinoic acid
Fam19a3	1.87	positive regulation of microglial cell activation	negative regulation of microglial cell activation
Naa11	−3.83	n-terminal protein amino acid acetylation	n-terminal peptidyl-glutamic acid acetylation
Rnf11	−1.75	protein autoubiquitination	ubiquitin-dependent protein catabolic process

### 2.4 Immunohistochemistry & semiquantitative image analysis

At each of four timepoints (P30, P90, P120 and >P150), Pde6b^wt/wt^ and Pde6b^rd1/rd1^ mice (n = 3 per genotype, unless otherwise indicated) underwent cervical dislocation before immediate enucleation and processing of retinae for IHC (supplementary methods). Images were taken at a point three fields of view (at ×40 magnification) from the ora serata in three sections from one eye in each animal. Co-localised staining for the proteins of interest and a bipolar cell marker were taken as a semiquantitative index of protein expression levels in bipolar cells and were determined using Costes' method, described and validated previously (Costes et al., 2004). In brief, this involves normalising the number of pixels demonstrating colocalisation above threshold in each image to the number of non-zero pixels in the image to give an index of colocalisation that could be compared between images (supplementary methods). A two-way ANOVA with Sidak's method to account for multiple comparisons between groups and Tukey's test for comparison with groups over time.

## 3 Results

### 3.1 Gene array comparison

Retinal ON-bipolar cells were isolated using FACS of dissociated retina from L7.Cre.EYFP transgenic mice which were additionally either wild type or homozygous for a clinically relevant IRD mutation (Pde6b^wt/wt^ or Pde6b^rd1/rd1^). cDNA libraries were extracted from the resulting EYFP+ (ON-bipolar) cell isolates were processed and subjected to an Illumina® mouse gene array. To quantify contamination of these isolates with rod photoreceptors, qPCR for two rod specific genes (Cnga1, Rho, chosen a priori) was performed to ensure that neither was detectable at a significant level in any bipolar cell isolate (Cnga1 – undetectable, Rho <1% in YFP + isolate compared to YFP -, Supplementary methods). Gene array analysis revealed sixty-six probes corresponding to sixty genes were shown to have differential expression between the degenerate and non-degenerate samples with a p value of <0.05 (False detection rate (FDR) testing, Fig. 1, Tables 1 and S2, supplementary methods).

### 3.2 Candidate genes

Following this, a sequence of methods was employed to highlight the most relevant genes, beginning with pathway analysis and database searches to highlight groups of genes with common function. Protein-protein interaction prediction software (Warde-Farley et al., 2010) highlighted one common function (“transition metal ion binding”) linking five of the differentially expressed genes (Atox1, Clip1, Msrb2, Mt1 & Mt2) involved in preventing and repairing oxidative damage. This was reinforced by use of the Reactome® knowledge base (Fabregat et al., 2016), demonstrating two similar overrepresented pathways (“Metallothionein metal binding” p = 0.009 & “Response to metal ions” p = 0.013) involving similar genes (Msrb2, Mt1 & Mt2). Searches of the RetNet database (Daiger et al., 1998) revealed only one differentially expressed gene (Srm2) to be implicated in human retinal disease.

Genes known to be related to the native bipolar cell light signalling pathway (Grm6 +1.054, p = 0.8573; Gnao1 -1.23, p = 0.9835; Gnb5 -1.13, p = 0.9670; Gng13–1.01, p = 0.7109; Trpm1 +1.19, p = 0.8035 (log_2_ fold change, adjusted p value, FDR, Table S3, Fig. S3) were specifically queried, and while highly expressed in absolute terms, none were significantly differentially expressed between groups. Similarly queried were genes more generally implicated in cell signalling (Table S3) and remodelling (e.g. glutamate, glycine and GABA receptors, Table S4) with no significantly differentially expressed genes identified.

As these methods revealed only one unifying functional pathway - “metal ion binding” (supplementary results) – this data driven approach was complemented by a manual, systematic, evaluation (including literature and gene ontology consortium annotation review) for each differentially expressed gene. This was used to group the potential candidates by function (Harris et al., 2004) and score them for relevance to our research question (supplementary methods). The highest scoring potential candidate in each group was selected for further investigation at the protein level using IHC: Srm2, coding for a gene involved in cell shape regulation, Slf2 heparin proteoglycan metabolism, Anxa7, neurotransmitter release and Cntn1 in synaptic remodelling (see Table 1).

### 3.3 Immunohistochemical staining

**Fig. 2:**
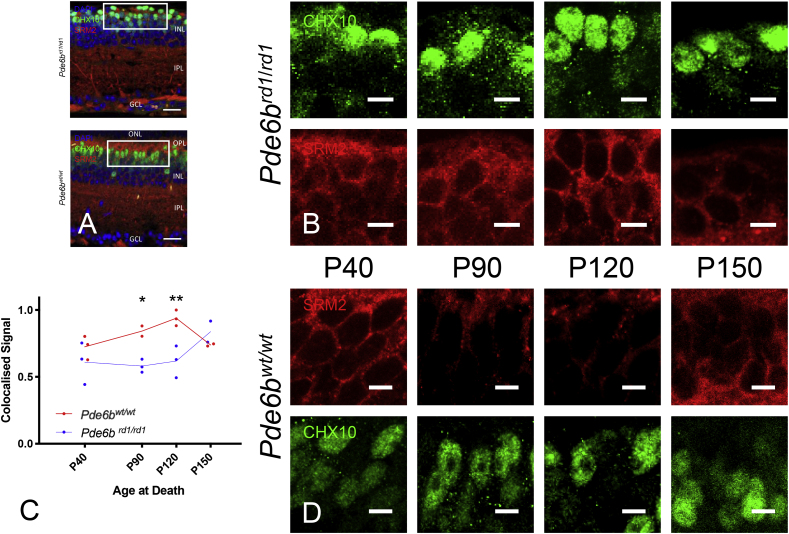
Shroom 2 Immunohistochemistry (upregulated on gene array at P90). (A) Retinal cross sections from Pde6b^rd1/rd1^ and Pde6b^wt/wt^ mice stained for DAPI (blue), CHX10 (green) and Shroom2 (red). ONL – Outer nuclear layer, OPL – Outer Plexiform layer, INL- Inner nuclear layer, IPL –inner plexiform layer, GCL – Ganglion cell layer. Scale bar = 20 μm. White box indicates area from which close up images in panels B & D are taken. (B&D) Close up images of bipolar cell bodies in Pde6b^rd1/rd1^ retina (B) and Pde6b^wt/wt^ (D) at four time points during degeneration (P40, P90, P120, P150). CHX10 (green) and shroom 2 (red). Scale bars = 5 μm. (C) Colocalised pixels above threshold - a semiquantitative index of protein staining. Normalised to highest value over all retinae stained for shroom 2. Dots represent the mean value for each animal, lines connect the means for each group at that timepoint. Star markers on graph represent adjusted p values, Sidak's method for multiple comparison (see results section for details): P90 p = 0.0136; P120 p = 0.0026 red line = Pde6b^rd1/rd1^; blue line = Pde6b^wt/wt^. (For interpretation of the references to colour in this figure legend, the reader is referred to the Web version of this article.)

**Fig. 3:**
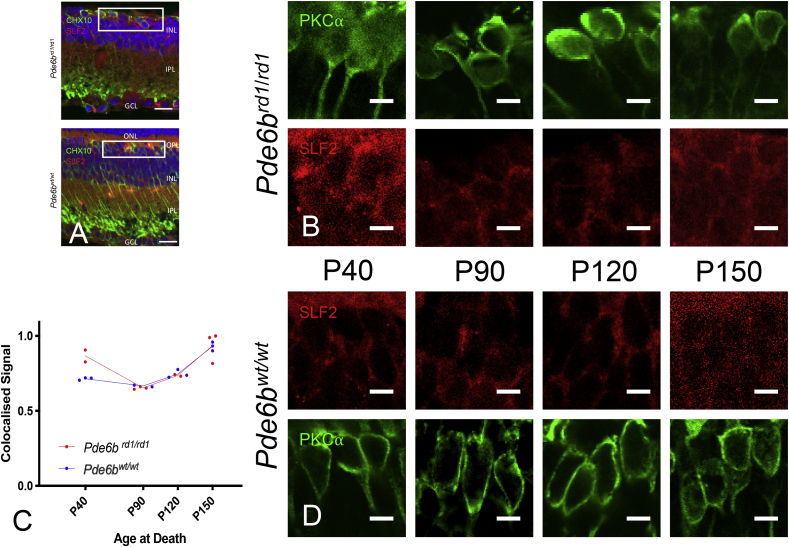
Sulphatase 2 Immunohistochemistry (upregulated on gene array at P90). (A) Retinal cross sections from Pde6brd1/rd1 and Pde6bwt/wt mice stained for DAPI (blue), PKC⍺ (green) and sulphtase 2 (red). ONL – Outer nuclear layer, OPL – Outer Plexiform layer, INL- Inner nuclear layer, IPL –inner plexiform layer, GCL – Ganglion cell layer. N.B. Due to primary antibodies being raised in differing species, to allow co-staining of sections, PKC⍺ is used as an ON-bipolar marker here, rather than CHX10. Scale bar = 20 μm. White box indicates area from which close up images in panels B & D are taken. (B&D) Close up images of bipolar cell bodies in Pde6brd1/rd1 retina (B) and Pde6bwt/wt (D) at four time points during degeneration (P40, P90, P120, P150). PKC⍺ (green) and Sulphatase 2 (red). Scale bars = 5 μm. (C) Colocalised pixels above threshold - a semiquantitative index of protein staining. Normalised to highest value over all retinae stained for Sulphatase 2. Dots represent the mean value for each animal, lines connect the means for each group at that timepoint. Star markers on graph represent adjusted p values, Sidak's method for multiple comparison (i.e. None reaching significance, see results section for details). red line = Pde6brd1/rd1; blue line = Pde6bwt/wt. (For interpretation of the references to colour in this figure legend, the reader is referred to the Web version of this article.)

**Fig. 4:**
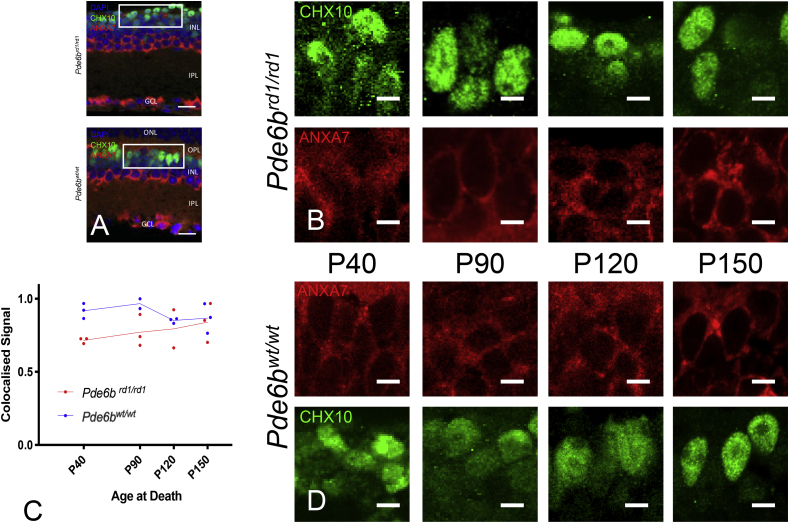
Annexin a7 Immunohistochemistry (downregulated on gene array at P90). (A) Retinal cross sections from Pde6b^rd1/rd1^ and Pde6b^wt/wt^ mice stained for DAPI (blue), CHX10 (green) and annexin a7 (red). ONL – Outer nuclear layer, OPL – Outer Plexiform layer, INL- Inner nuclear layer, IPL –inner plexiform layer, GCL – Ganglion cell layer. Scale bar = 20 μm. White box indicates area from which close up images in panels B & D are taken. (B&D) Close up images of bipolar cell bodies in Pde6b^rd1/rd1^ retinae (B) and Pde6b^wt/wt^ (D) at four time points during degeneration (P40, P90, P120, P150). CHX10 (green) and annexin a7 (red). Scale bars = 5 μm. (C) Colocalised pixels above threshold - a semiquantitative index of protein staining. Normalised to highest value over all retinae stained for annexin a7. Dots represent the mean value for each animal, lines connect the means for each group at that timepoint. Star markers on graph represent adjusted p values, Sidak's method for multiple comparison (i.e. None reaching significance, see results section for details). red line = Pde6b^rd1/rd1^; blue line = Pde6b^wt/wt^. (For interpretation of the references to colour in this figure legend, the reader is referred to the Web version of this article.)

**Fig. 5:**
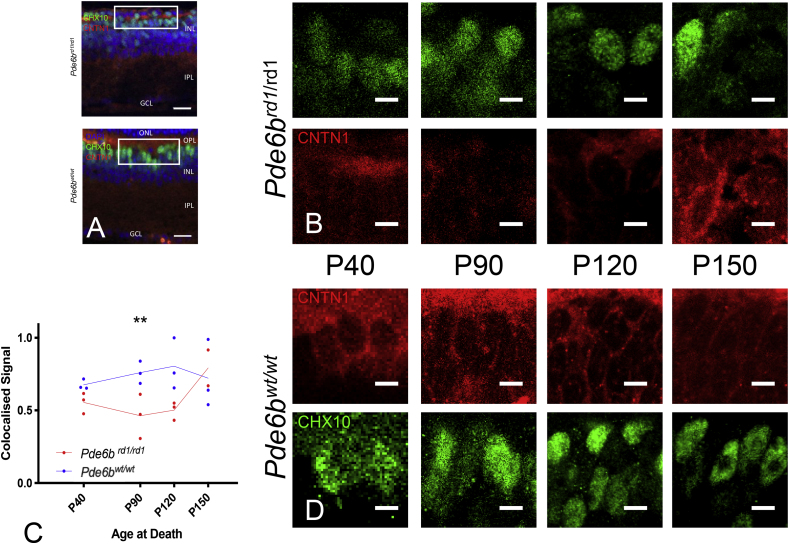
Contactin1 Immunohistochemistry (downregulated on gene array at P90). (A) Retinal cross sections from Pde6b^rd1/rd1^ and Pde6b^wt/wt^ stained for DAPI (blue), CHX10 (green) and Contactin 1 (red). ONL – Outer nuclear layer, OPL – Outer Plexiform layer, INL- Inner nuclear layer, IPL – inner plexiform layer, GCL – Ganglion cell layer. Scale bar = 20 μm. White box indicates area from which close up images in panels B & D are taken. (B&D) Close up images of bipolar cell bodies in Pde6b^rd1/rd1^ retina (B) and Pde6b^wt/wt^ (D) at four time points during degeneration (P40, P90, P120, P150). CHX10 (green) and Contactin 1 (red). Scale bars = 5 μm. (C) Colocalised pixels above threshold - a semiquantitative index of protein staining. Normalised to highest value over all retinae stained for Contactin 1. Dots represent the mean value for each animal, lines connect the means for each group at that timepoint. Star markers on graph represent adjusted p values, Sidak's method for multiple comparison at P90 p = 0.0026) (see results section for details). red line = Pde6b^rd1/rd1^; blue line = Pde6b^wt/wt^. (For interpretation of the references to colour in this figure legend, the reader is referred to the Web version of this article.)

Antibody labelling for proteins encoded by each of the selected genes (Srm2, Slf2, Anxa7, Cntn1) showed co-localisation with classical ON bipolar markers (CHX10 or PKCa) at postnatal day 90 (P90) in our IHC study – although these proteins were typically not expressed exclusively within ON BCs (Fig. 2, Fig. 3, Fig. 4, Fig. 5). A semi-quantitative index of staining was recorded for each genotype (Pde6b^wt/wt^ & Pde6b^rd1/rd1^) at each timepoint (P40, P90, P120, P150) to give an impression of how protein levels may change over time.

There was a difference between genotypes in this index for all proteins excepting slf2 (Sulphatase 2) [Srm2 F (1, 8) = 23.85; p = 0.0012, Slf2 F (1, 8) = 3.775; p = 0.0879, Anxa7 F (1, 9) = 5.31; p = 0.0467; Cntn1 F (1, 13) = 8.272; p = 0.0130]. In addition, differences in staining over time were seen in Srm2 (Shroom 2) & Slf2 [Srm2 F (3, 8) = 4.865; p = 0.0327, Slf2 F (3,8) = 19.9; p = 0.005, Anxa7 F (3, 9) = 0.1829; p = 0.9053, Cntn1 F (3, 13) = 0.168; p = 0.9161]. Whilst an interaction between genotype and time was seen in Srm2 and Cntn1 (Contactin 1) [Srm2 F (3,8) = 0.09; p = 0.0059, Slf2 F (3,8) = 2.718; p = 0.1148, Anxa7 F (3,9) = 0.6712; p = 0.5909, Cntn1 F (3,13) = 7.366; p = 0.0039]. (Please see Fig. 2, Fig. 3, Fig. 4, Fig. 5 and supplementary material for post hoc analysis).

## 4 Discussion

### 4.1 Bipolar cells

Much of mammalian basic image processing is initiated within the neural retina before signals reach retinorecipient visual centres. As vision is lost in the IRDs through preferential photoreceptor (PR) death, the retinal bipolar cells become the highest surviving cells of this neural hierarchy. As such, their stimulation may - in principle - enable more intra-retinal processing to be preserved at the synapses of the inner plexiform layer, presenting them as particularly attractive targets for optogenetic visual restoration.

Targeting bipolar cells assumes that, within a degenerate retina, these cells retain levels of the second messengers required for light signalling. It also assumes such cells survive in a stable state without metabolic stress to allow effective propagation of this signal by synaptic neurotransmitter release and, importantly, are able to be transduced by AAV. Increasing evidence confirms that bipolar cells do undergo significant remodelling in the later stages of retinal degeneration with changes in morphology, synaptic connections, electrophysiological responses and receptor expression (Cuenca et al., 2014; Dunn, 2015; Gayet-Primo and Puthussery, 2015; Kalloniatis et al., 2016; Marc and Jones, 2003; Marc et al., 2007; Michalakis et al., 2013; Strettoi et al., 2002).

### 4.2 Gene expression in context

Here we show that, despite this remodelling, bipolar cells undergo remarkably limited transcriptomic changes in response to the loss of synaptic inputs from photoreceptors, even in the late stages of the disease. While gene array studies of whole retina may not have statistical power to detect very small changes in expression of single genes, our approach of limiting our comparison as far as possible to a single cell type in a clinically relevant disease model, will accentuate those changes that are biologically most relevant.

Given the marked changes seen at the anatomical level over the whole retina during the neural remodelling of degeneration, it is perhaps surprising that we found such a small number of genes were differentially expressed in bipolar cells in this context (66 out of a total of c.20,000 probes). The absence of differential expression of genes related to the native bipolar cell light signalling cascade, second messaging in general or glutaminergic transmission (Tables S3 and S4) is particularly interesting given reports of functional loss of sensitivity to glutamate even early in degeneration (Varela et al., 2003). We see no significant alteration in expression of genes associated with glutamate receptor subunits (nor with GABAergic, nor glycinergic receptors, Table S4) which is intriguing given the shift from metabotropic to ionotropic transmission seen at a functional and anatomic level in bipolar cells during degeneration (Dunn, 2015; Marc et al., 2007; Varela et al., 2003).

This finding of such stability at a gene expression level is particularly informative when seen in the light of studies where opsin based optogenetic tools (Cehajic-Kapetanovic et al., 2015; De Silva et al., 2017; Lin et al., 2008) are targeted to ON-bipolar cells to functionally restore light responses in degenerate retina. Therefore, despite marked functional and anatomical remodelling, the parts of the bipolar cell signalling cascade necessary for optogenetic restoration appear to persist both at a gene expression and functional level during retinal degeneration in the Pde6b^rd1^ model. Given that the rd1 mutation causes an IRD in humans similar in phenotype to that of the model, these findings are particularly interesting from a translational point of view (if they are reflected in human bipolar cells). In counterpoise however, the huge variety of causative mutations in human IRDs should still be borne in mind when extrapolating results.

### 4.3 Identifying candidate genes

With a relatively small number of differentially expressed genes overall and no obvious candidate genes presented for further investigation by data driven approaches (supplementary results), a systematic literature review for each gene could be used to group and prioritise those most promising for further characterisation at the protein level (Srm2, Slf2, Anxa7, Cntn1). Immunohistochemical staining at the timepoint corresponding to the gene array (P90) confirmed expression of all four proteins in bipolar cells of both Pde6b^wt/wt^ and Pde6b^rd1/rd1^ retinae. Staining of similar retinae at other timepoints over the course of degeneration (Fig. 2, Fig. 3, Fig. 4, Fig. 5, Table 2) could be additionally analysed in a semi-quantitative manner to given an impression of likely relative protein expression over time in order to guide potential future investigations.

**Table 2:** Details of four differentially expressed genes prioritised for further characterisation with IHC. The bottom four rows refer to semiquantitative IHC co-localisation seen in Fig. 2, Fig. 3, Fig. 4, Fig. 5 p = - “adjusted p value”; n.s. – p > 0.05.

Gene	Srm2	Slf2	Anxa7	Cntn1
Protein	Shroom 2	Sulphatase 2	Annexin a7	Contactin 1
Function	Actin cytoskeleton, axon guidance	HSPG metabolism	Membrane fusion, neurotransmitter release	Neuron projection development, synaptic remodelling
Comments	Regulates actin cyctoskeleton and therefore cell shape, axon sprouting and organelle location (essential for viral transduction, second messenger systems and neurotransmitter release) (Fairbank et al., 2006).	Extracellular endosulphatase. Removes sulphate residues from cell surface HSPG residues (Morimoto-Tomita et al., 2002) - important in the entry of AAV into cells (Summerford and Samulski, 1998) as well as retinal synaptic plasticity	Calcium dependant phospholipid binding protein implicated in synaptic neurotransmitter release and the bipolar cell light response (Caohuy and Pollard, 2002; Grewal et al., 2016; Hoque et al., 2014). PKCα (important in activation and termination of bipolar cell light response phosphorylates annexin a7 promoting membrane fusion and so neurotransmitter release (Hoque et al., 2014). PKCα expression was not altered in our gene array comparison.	A cell surface glycoprotein implicated in synaptic plasticity (Davisson et al., 2011)
Human disease caused defect in gene	Implicated in retinal degeneration with deafness (Fairbank et al., 2006)	Not reported	Not reported	Congenital Myopathy (Davisson et al., 2011)
Mouse knock outs	Knock down - failure of retinal lamination, full knock out - no retinal phenotype. Other Srm family members may compensate. (Fairbank et al., 2006)	Deficiencies in neural remodelling; no major developmental flaws or retinal defects reported (Masu, 2013)	No gross neurological phenotype, retina not examined (Grewal et al., 2016)	Impaired synaptic long-term depression (LTD) (Murai et al., 2002). Over-expression improves long term potentiation (LTP) (Gulisano et al., 2017). No retinal phenotype at P14 (Chatterjee et al., 2019)
Expression at P90 (gene array)in Pde6b^rd1/rd1^vs Pde6b^wt/wt^ (FDR adj’ p value)	Up (p = 0.042)	Up (p = 0.034)	Down (p = 0.024)	Down (p = 0.049)
Expected location of protein within cell	Cell membrane (Etournay et al., 2007) confirmed in cell culture (Fig. S1) & IHC (Fig. 2)	Cell surface, extracelluar (Morimoto-Tomita et al., 2002) Confirmed in cell culture (Fig. S1) & IHC (Fig. 3)	Cell membrane (Watson et al., 2004) confirmed in cell culture (Fig. S1) & IHC (Fig. 4)	Cell surface (Davisson et al., 2011), confirmed in cell culture (Fig. S1) & IHC (Fig. 5)
Previous IHC of Pde6b^wt/wt^ retinae	Retinal pigment epithelium, bipolar cell bodies, inner plexiform layer (Etournay et al., 2007)	Bipolar cell bodies, outer plexiform layer photoreceptors synapses (Orlandi et al., 2018)	Not described previously in retina	Bipolar cell and outer plexiform layer (Davisson et al., 2011)
2-way ANOVA – difference by genotype (Pde6b^rd1/rd1^vs Pde6b^wt/wt^)	Yes F (1, 8) = 23.85; p = 0.0012	No F (1, 8) = 3.775; p = 0.0879	Yes F (1, 9) = 5.31; p = 0.0467	Yes F (1, 13) = 8.272; p = 0.0130
2-way ANOVA – difference by time	Yes F (3, 8) = 4.865; p = 0.0327	Yes F (3,8) = 19.9; p = 0.005	No F (3, 9) = 0.1829; p = 0.9053	No F (3, 13) = 0.168; p = 0.9161
2-way ANOVA - interaction genotype x time	Yes F (3,8) = 9.09; p = 0.0059	No F (3,8) = 2.718; p = 0.1148	No F (3,9) = 0.6712; p = 0.5909	Yes F (3,13) = 7.366; p = 0.0039
Significant Post Hoc Tests	Sidak P90 – p = 0.0136; p120 - p = 0.0026 Tukey Pde6b^rd1/rd1^:P40 vs P150 p = 0.0307; P90 vs P150 p = 0.0157; Pde6b^wt/wt^:P40 vs P120 p = 0.0126); P120 vs P150 p = 0.0199	Tukey Pde6b^rd1/rd1^:P40v s P90 p = 0.0083; P90 vs P150 p = 0.0028; P120 to P150 p = 0.0248; Pde6b^wt/wt^: P40 vs P150 p = 0.0146; P90 vs P150 p = 0.0052; P120 vs P150 p = 0.0116	Sidak Not significant at any individual timepoint	Sidak P90 p = 0.0026

### 4.4 Further characterisation of selected candidate genes

The pattern of Shroom 2 staining (Fig. 2) that we see in degeneration, with a maxima in mid degeneration (where neural modelling is at its highest), is consistent with its described role in cytoskeleton remodelling, cell shape regulation and membrane blebbing, given the retraction of bipolar cells axons and change in shape seen in histological studies of degeneration (Jones and Marc, 2005; Strettoi et al., 2002). A corresponding upturn in staining at P150 in wild type animals was seen in multiple replicates and could perhaps be explained by an increase in neural remodelling in older mice – which would certainly be an interesting target for further investigation. In the broadest terms, this may suggest intervention earlier in the course of degeneration may be beneficial whilst cytoskeleton and membrane activity (such as AAV entry, payload trafficking and neurotransmitter release) are possibly less disrupted.

The Heparin Sulphate Proteoglycans (HSPG), from which Sulphatase 2 removes sulphate residues, are involved in (but not essential to) the binding of AAV in advance of its entry to the cell (Summerford and Samulski, 1998). Importantly, AAVs are less able to bind HSPGs that are less sulphonated (for example due to increased sulphatase activity) and indeed HSPGs have been found to be functionally important in retinal cell transduction efficiency, especially by the intravitreal route (Boye et al., 2016; Woodard et al., 2016). Interestingly, our semiquantitative IHC (Fig. 3), unlike gene array data, suggests no difference in Sulphatase 2 staining compared to wild type during degeneration. However, this could represent an increase in protein turnover (and hence RNA levels), a shift to the secreted, extracellular form of the protein during degeneration (Morimoto-Tomita et al., 2002) from the cell surface bound sulphatase 2 or indeed post-transcriptional changes at the mRNA level and so gene expression levels do not directly reflect the level of cell staining. Therefore, it may be fruitful to investigate quantitatively HSPG sulphonation in various cell types of the degenerate retina, compared to wild type. Or indeed, if changes in Slf2 levels can manipulate AAV transduction efficiency (for example by investigating AAV transduction in a Slf2 knock out retinae (Table 2).

Effective retinal optogenetic therapy requires neurotransmitter release from targeted cells; Annexin a7 is central to this process and interacts with PKCα, an enzyme known to regulate bipolar cells' light response kinetics (Table 2) (Hoque et al., 2014) (supplementary discussion). Anxa7 IHC staining in our series is just significantly different from wild type during degeneration (but with no individual timepoint identified as significant on post hoc analysis), this could suggest that this aspect of the bipolar signalling cascade is indeed still functional, but with a reduced rate of protein turn over when light signalling is lost during retinal degeneration. A more comprehensive understanding of the role of Anax7's in the wild type bipolar cell light response will need to be determined if any downregulation is likely to directly impair bipolar cells ability to act as optogenetic targets, or indeed represent an opportunity to manipulate response kinetics.

To act as optogenetic targets, bipolar cells must not only be able to release neurotransmitter, but to maintain useful synapses to communicate the transduced light signal. Contactin 1 has a role in regulating synaptic plasticity in the nervous system, so our finding that it was downregulated at P90 in retinal degeneration – a process defined by neural remodelling (Jones and Marc, 2005) - was perhaps surprising. The transient drop in IHC staining (at P90 only) in our series (Fig. 5 and Table 2) is however congruent with the findings of Haenisch et al. who also noted an initial marked decrease of neural Cntn1 mRNA expression followed by an increase back to baseline when investigating nerve crush (deafferention) in zebrafish (Haenisch et al., 2005). Which, in isolation, could perhaps predict a benefit to early optogenetic intervention in the retina, restoring afferent signal input in an attempt to prevent a drop in Contactin 1 and any resulting maladaptive synaptic remodelling.

### 4.5 Limitations

While representing the first description of transcriptomic changes in bipolar cells in the context of degenerative retina remodelling, there are limitations to our approach that must be borne in mind when extrapolating results.

Firstly, as alluded to above, we predicted that rod photoreceptors may be a contaminant of our cell isolates and therefore assessed sample purity in two ways – by interrogating our microarray samples for expression of genes known to be specific for rods and by performing qPCR for a small number of these markers. However we did not quantify markers specific to other retinal cell types to absolutely exclude contamination from other populations.

Secondly, microarrays incorporate multiple technical controls and have been shown to faithfully replicate the mRNA quantification results of other methods (such as qPCR) (Arikawa et al., 2008; Canales et al., 2006; Morey et al., 2006) and in meta-analysis studies show concordance across array platforms (Brown et al., 2017). Therefore, we did not employ alternative methods to externally validate our microarray findings at the mRNA level, but rather, investigated a subset of candidate genes by IHC. This approach provided a wealth of additional information on both the spatial distribution within the retina as well as whether mRNA changes actually affected protein levels. However, mRNA modifications, transport and post translational modifications could potentially occur. Indeed, further investigation of such processes will form an important future direction of investigation – especially as they may explain the lack of change in staining for Slf2 & Anxa7 at P90.

Thirdly, as this is the first study to investigate transcriptional changes in on-bipolar cells during retinal degeneration, a direct positive-control - a gene already known to be up or down regulated in this context – was lacking. Such positive controls provide a valuable technical validation of transcriptomic datasets but were not possible in this context.

## 5 Conclusion

Here we present the first comparison of gene expression in bipolar cells of degenerate and non-degenerate retinae. Our findings suggest relatively few changes in gene expression with degeneration, including genes essential to effective optogenetic bipolar light signalling. This suggests, that despite remodelling, bipolar cells are likely to remain viable and effective targets for optogenetic vision restoration and we highlight candidate genes where further investigation is likely to improve the translation of this important technique.

## Data Availability

Microarray data has been uploaded to the EMBL-EBI ArrayExpress platform and is available with the accession number E-MTAB-10357.

## Funding

This work was supported by The 10.13039/100010269Wellcome Trust (grant number 205151/Z/16/Z); The Woolf Fisher Trust; The 10.13039/501100001659German Research Foundation (DFG) (grant number LI2846/1-1) and The Biological Basic Sciences Research Council (grant number BB/M009998/1).

## Declaration of competing interest

M.L. reports non-financial support from Optos, Genentech/Roche and Heidelberg Engineering, personal fees from Alimera Sciences, outside the submitted work.M.J.G., D.H., S.H., S.N.P., R.E.M. and M.W.H. have no relavent financial disclosures.

## Statement

Aspects of this work were presented at the 2018 Association for Research in Vision and Ophthalmology annual meeting at Honolulu, Hawaii, USA.
