# Supplementary material for: ON-bipolar cell gene expression during retinal degeneration: Implications for optogenetic visual restoration
Source: Exp Eye Res. 2021 Jun;207:108553. doi: 10.1016/j.exer.2021.108553 (PMC8214074; doi:10.1016/j.exer.2021.108553)
Supplement: Multimedia component 1 [file mmc1.docx]

# Supplementary Material

## Supplementary Methods

### Housing and validation of mouse line

Mice were housed under twelve-hour light / dark cycle at 21^o^C, diet and water were available ad libitum. The transgenic murine model (“L7.Cre.EYFP.*Pde6b*^x/x^”) was created by crossing and selectively breeding three existing lines:

1. Tg(Pcp2-cre)756Mro/0 (Silvia Marino, 2002), a gift from Prof. Sebastian Brandner, UCL
2. Gt(ROSA)26Sor^tm1(EYFP)Cos^ mice(Srinivas et al., 2001), a gift from Elizabeth Slee, University of Oxford
3. C3H/HeN *Pde6b*^rd1/rd1^(Pittler and Baehr, 1991)

Mice were confirmed to be wildtype for *Gpr179^Nob^* allele(Nishiguchi et al., 2015) and as demonstrated in *Figure S2*, *L7.Cre.EYFP.Pde6b^rd1/rd1^* mice were seen to both appropriately express EYFP in their ON-bipolar cells and also demonstrate a retinal degenerate phenotype in keeping with previous histological descriptions of *Pde6b*^rd1/rd1^ homozygotes (Bowes et al., 1990).

### FACS cell isolation and validation

*MoFlo Legacy* with associated *Summit* software (both Beckman Coulter, Brea USA) was used to isolate EYFP labelled cells from both *Pde6b^wt/wt^* and *Pde6b^rd1/rd1^* groups. Excitation was provided by a 488nm laser set to 100mW (Sapphire 488-200, Coherent, Santa Clara, USA).

The identity of the sorted samples was confirmed by immunocytochemistry (ICC) with EYFP marker + cells seen to co-stain with the bipolar cell marker PKCα. Quantitative polymerase chain reaction (qPCR) using standard methods(Peirson, 2007) demonstrated a fifteen-fold increase in the EYFP+ isolate compared to the EYFP- for bipolar cell specific genes (*Cabp5, Grm6, Pcp2 and Prkca*) when normalised to housekeeping genes (*Gapdh, Actb, Rplp0*). As these genes are extensively expressed in cell processes, this upregulation compared to the isolate of YFP negative cells would suggest bipolar cell processes are not lost completely during digestion (but cannot confirm this in itself). Rod specific gene (*Rho & Cnga1*) expression in the EYFP+ isolate less than 1% of that in the EYFP- isolate.

### RNA extraction, cleaning & amplification

Sorted cells were stored in RLT (lysis) buffer with 2-mercaptoethanol and RNA extraction immediately proceeded to using an RNAeasy micro kit (Qiagen, Germantown, USA), according to the manufacturer’s instructions. Extracted RNA from the EYFP+ cell isolates (*Pde6b*^wt/wt^ and *Pde6b*^rd1/rd1^) was processed for use on a *Mouse WG-6* microarray (Illumina, San Diego, CA).

RNA quantity and purity were first assessed using a *2100 Bioanalyzer* (Agilent Technologies, Santa Clara, USA) with a mean RNA concentration of 364±143 pg/ul; RIN 9.5±0.1 in *Pbe6b*^rd1/rd1^ retinae and 608±263 pg/ul; RIN 9.4±0.2 in *Pbe6b*^wt/wt^.

Total RNA was amplified using an *Ovation Pico WTA v2* kit (NuGEN Technologies, San Carlos, CA). Amplified cDNA was biotinylated using a *BiotinIL* kit (NuGEN Technologies) before being hybridised to a MouseWG-6 v2 Expression BeadChip (Illumina).

The BeadChip was read with a Bead Array Reader (Illumina) and gene array data imported to GenomeStudio (Illumina) and processed using Lumi(Du et al., 2008) and Limma(Ritchie et al., 2015) packages for R(Team, 2013). Probes with fluorescence values not significantly different (*P* > 0.01) from control were excluded and quantile normalization was used to remove technical variation between arrays. Results were corrected for multiple testing using False Discovery Rate (FDR) testing(Benjamini, 1995). Probes were considered to be differentially expressed when adjusted *p* < 0.05.

Quality controls integrated into the arrays included hybridization controls; mismatch and perfect match probes for stringency; labelling controls and negative controls. All control results were within expected limits. Biological replicates are described in the methodology section and repeated below (MIAME summary)

### Probes known to be related to the bipolar light signaling cascade

Probes for genes specifically annotated as: *“g-protein coupled receptor subunits,” “g-protein associated kinases,” “arrestins,” “transient receptor potential M channels” and “neurotransmitter secretion”* were specifically interrogated to identify any differentially expressed gene that may be impact on the principle native light signal through bipolar cells (Table S3). Five genes, representing important aspects of the known ON-bipolar cell light signal transduction cascade were specifically interrogated *Grm6, Gnao1, Gnb5, Gng13, Trpm1.* None of these probes were returned as significantly different.

### Gene ontology database search and systematic literature review

Search terms:

NCBI “Gene” database:

(Gene name[gene]) AND (Mus musculus[orgn])

Medline ®

(((Gene name[Title/Abstract]) AND retina[Title/Abstract]) AND ("1900"[Date - Publication] : "3000"[Date - Publication])) AND "english"[Language]

Initially titles were reviewed and genes with no relevant literature identified and removed from further investigation. Review of abstracts then allowed primary literature relevant to each gene’s retinal expression to be obtained and reviewed. Within each group of related ontology annotations, primary literature was used to guide prioritisation of a genes for initial further investigation to act as an exemplar. This ranking was based on a subjective assessment of the primary literature to give a score from five in each of three domains:

1. The likelihood of involvement with the primary light signal transduction cascade in bipolar cells
2. Potential clinical implications
3. Actions in retinal remodelling

The gene with the highest rank score out of 15 in each group was taken forward for further investigation.

As the changes in group E “Aerobic and anaerobic respiration, cellular responses to stress” were as expected in a retina of a P90 *Pde6b*^rd1/rd1^ mouse (a downregulation of genes involved in aerobic glycolysis and prevention of oxidative stress) and this group least directly impinges on the propagation of the native light signal, a GOI was not chosen from this group for initial validation.

### Immunohistochemistry

Directly after enucleation, eyes were transferred to 4% PFA for twenty-four hours before incubation in 30% sucrose phosphate buffered saline (PBS) for a further forty-eight hours to cryoprotect. The eyes were then embedded in optimal cutting temperature (OCT) solution and 20μm sections captured onto poly-lysine coated microscopy slides. Immunohistochemistry & Immunocytochemistry was performed on retinal sections using previously published methods(De Silva et al., 2017) with the addition of an initial incubation at 95^o^C for sixty minutes with 10 mmol/l Sodium citrate, 0.05% Tween 20, pH 6.0 as an antigen retrieval step.

### Immunohistochemistry Antibody selection and validation

The antibodies detailed on *Table S5* were selected and validated by performing ICC on cultured human embryonic kidney cells (HEK293T) transfected with the appropriate plasmid, according to published methods(Hughes et al., 2015) *(Figure S1)*. This method demonstrates that the described antibodies stain specifically for the expected proteins in an in vitro system. Retinal tissue from knock out animals could be a useful further validation if these candidate genes are taken forward to functional validation.

### Immunohistochemistry: index of co-localisation

Using a Zeiss LSM 710 Observer® confocal microscope (Zeiss, Oberkochen, Germany), a 10 frame Z-stack image spanning the depth of the entire retinal section was taken using a x40 objective at a point three fields of view from the ora serrata with consistent gain and power settings. These images were then analysed using Image J® (Schindelin et al., 2012) and the plugin JACOP(Bolte and Cordelieres, 2006) which was used to apply the methodology of Costes et al (Costes et al., 2004) to return an index of co-localisation. These values were then normalised within the replicates of each protein stained for, against the highest value in that group.

## Supplementary Results

### GOI identification

Searches of the Medline® & gene ontology databases and systematic literature review revealed nine candidate genes (*Gm5478, D730001G18, Fam95a, Gm362, Sfxn1, Ccdc171, Stmnd1, Csrnp2 & Igfn1*) to have no relevant literature; nine more to have an ontological annotation and literature indicating rod specificity (*Pdc, Pde6b, Gnat1, Nrl, Sag, Slc24a1, Pde6g, Reep6, Esrrb*) (see discussion). Conversely, a further five (*Gpnmb(Bächner et al., 2002), Ldha [*Lasson et al*], Car2(Vardimon et al., 1986), Adcy1*(Hwang et al., 2013)*, Pde9a(Dhingra et al., 2014)* already had descriptions of their retinal cell protein expression suggesting contentiousness (*Table S1*).

### Immunohistochemistry

Immunoreactivity of each antibody at or around the membrane in cells labelled with a bipolar marker (CHX10 or PKCα) antibody was demonstrated on sections from mice sacrificed at P90 (*Figures 2-5*). As expected, all four proteins of interest were seen to be present in or around the membrane. This colocalisation was reinforced by image analysis with all proteins of interest (POI), timepoints and repetitions demonstrating an ICQ value(Li et al., 2004) between 0 and 5 - indicating colocalisation of staining for POI and bipolar cell markers.

Colocalised pixels above threshold at each timepoint gave a semi-quantitative index of protein levels (*Figures 2-5*). In general, this appeared to reflect the direction of differential expression (up or down) seen on the gene array, however this difference was only significant at P90 for Contactin 1 and Shroom 2.

### ANOVA of Immunohistochemical staining

*Here are included ANOVA results arranged by protein for ease of reference along with significantly different post-hoc tests (non-significant results have been omitted for brevity).*

*Srm2* (Shroom 2), this showed a difference between genotypes (F(1, 8) = 23.85; *p*=0.0012), and with time (F(3, 8) = 4.865; *p*=0.0327) and also an interaction between time and genotype (F(3,8) = 0.09; *p*=0.0059). Sidak’s method showed significantly higher staining in degenerate retinae at P90 (*p*=0.0136) and P120 (*p*=0.0026) see *figure* 2. In the *Pde6b^rd1/^*^rd1^ group, Tukey’s method demonstrated differences between P150 and both P40 (*p*=0.0307) and P90 (*p*=0.0157) with a decrease in staining as degeneration progresses. Staining in the *Pde6b^wt/^*^wt^ group at P120 was lower than at P40 (*p*=0.0126) and P150 (*p*=0.0199) giving a trough in staining at this point (*figure* 2).

For *Slf2* (Sulphatase 2), there was no difference between genotypes (F(1, 8) = 3.775 ; *p=*0.0879), there was a change with time (F(3,8) = 19.9; *p*=0.005), but no interaction between time and genotype (F(3,8) = 2.718; *p*=0.1148). In the *Pde6b^rd1/^*^rd1^ group, Tukey’s method demonstrated differences between P90 and both P40 (*p*=0.0083) and P150 (*p*=0.0028) as well as between P120 and P150 (*p*=0.0248) summarised as a decrease in staining in mid degeneration. In the *Pde6b^wt/^*^wt^ group, P150 differed from P40 (*p*=0.0146), P90 (*p*=0.0052) and P120 (*p*=0.0116) – showing increasing expression with age (*figure* 3).

For *Anxa7* (Annexin a7), there was a difference between genotypes, (F(1, 9) = 5.31; *p*=0.0467), but not with time (F(3, 9) = 0.1829; *p=*0.9053), nor was there an interaction between time and genotype (F(3,9) = 0.6712; *p*=0.5909). Sidak’s method for multiple comparisons did not show any significant difference between genotypes at any individual time point (*figure* 4) suggesting that Annexin a7 staining is constant over time in both groups.

With *Cntn1* (Contactin 1), there was a difference between genotypes (F(1, 13) = 8.272; *p=*0.0130), but not with time (F(3, 13) = 0.168; *p=*0.9161), there was an interaction between time and genotype (F(3,13) = 7.366; *p*=0.0039). Sidak’s method for multiple comparisons showed a significant difference between genotypes at P90 only (p=0.0026) with reduced expression in degenerate retinae at this timepoint (*figure* 5).

## Supplementary discussion

### Oxidative stress

Despite relative stability in gene expression profiles in bipolar cells between wild type and degenerate retina, changes in expression *were* evident in sixty genes and so pathway and protein-protein interaction analysis approaches were used. This aimed to discern any function common to groups of genes in order to assist in selecting candidates for further investigation with minimum bias. This returned a small number of common functions, relating to oxidative stress.

As photoreceptors are lost during retinal degeneration, their associated high aerobic metabolic demand and oxidative stress on surrounding tissue falls, reducing the need for energy and antioxidant proteins(Marc and Jones, 2003). Therefore, by P90 in the *Pde6b*^rd1/rd1^ retina, bipolar cells should no longer show signs of oxidative stress if they are to avoid activating the apoptotic pathways(Cuenca et al., 2014) and survive to act as optogenetic targets. In keeping with this, we see significant downregulation of genes associated with the respiratory chain and prevention of acute oxidative damage and upregulation of *Msrb2*, involved repair of oxidative damage(Pascual et al., 2010) suggesting that, as would be expected, by P90 bipolar cells are under reduced oxidative stress. As changes in these genes were as expected, we did not take them forward for further characterisation.

## Anxa7 & Unc13a

Effective optogenetic therapy requires effective neurotransmitter release from targeted cells and two genes central to this process appeared downregulated in degenerate retinae (*Anxa7 and Unc13a*). While expressed at bipolar cell “conventional synapses,(Schmitz et al., 2001)” UNC13A does not appear to be expressed at the main ribbon synapses bipolar cells use to propagate the light signal with great speed and efficiency, so is less likely to have a direct role in primary transmission in these particular cells. However, annexin a7 is known to interact with PKCα, central to the regulation of bipolar cells’ light response kinetics (*table 2*)(Hoque et al., 2014), so was selected for further characterisation.

# MIAME

MIAME describes the Minimum Information About a Microarray Experiment that is needed to enable the interpretation of the results of the experiment unambiguously and potentially to reproduce the experiment(Brazma et al., 2001). Our data is deposited in the Array Express database. The six cardinal elements of MIAME are listed below and with annotation on how they relate to this study:

- *The raw data for each hybridisation (e.g., CEL or GPR files)*
  - These will be deposited in the Array Express repository
- *The final processed (normalised) data for the set of hybridisations in the experiment (study) (e.g., the gene expression data matrix used to draw the conclusions from the study).*
  - These will be deposited in the Array Express repository
- *The essential sample annotation including experimental factors and their values (e.g., compound and dose in a dose response experiment).*
  - No drug was used in this study, the difference between groups was the presence or not of homozygosity for rd1. This is validated histologically and described in the text (*Figure S2*)
- *The experimental design including sample data relationships (e.g., which raw data file relates to which sample, which hybridisations are technical replicates, which are biological replicates).*
  - Biological replicates: As detailed in the methods section, YFP-positive cells were isolated by FACS from six non-degenerate (*Pde6b+/+*) and six degenerate (*Pde6brd1/rd1*) mice for a microarray study of bipolar cell gene expression changes in retinal degeneration. Each mouse represented one biological replicate. Both non-degenerate and degenerate groups each consisted of three males and three females. Two sibling pairs of the same gender were used, with one of the siblings being retinal degenerate and the other non- degenerate. The 12 mice were sourced from five breeding pairs. Mice were 89 to 91 days old on the day of FACS (Table 7.1). Non-degenerate retinas yielded 95,300 ± 10,900 YFP-positive cells and degenerate retinas yielded 73,200 ± 13,900 YFP-positive cells.
  - Technical replicates will be appropriately labelled when the data is uploaded to the repository.
  - These .csv files are available on the Array Express repository, appropriately labelled.
- *Sufficient annotation of the array (e.g., gene identifiers, genomic coordinates, probe oligonucleotide sequences or reference commercial array catalogue number).*
  - Please see methods sections; annotation files are available in the Array Express repository Illumina Mouse WG-6 v2 microarray
- *The essential laboratory and data processing protocols (e.g., what normalisation method has been used to obtain the final processed data).*
  - Please see methods & supplementary methods sections

# References

Bächner, D., Schröder, D., Gross, G., 2002. mRNA expression of the murine glycoprotein (transmembrane) nmb (Gpnmb) gene is linked to the developing retinal pigment epithelium and iris. Gene Expression Patterns 1, 159-165.

Benjamini, Y.H., Yosef, 1995. Controlling the false discovery rate: a practical and powerful approach to multiple testing. J Royal stat soc 57.

Bolte, S., Cordelieres, F., 2006. A guided tour into subcellular colocalization analysis in light microscopy. Journal of microscopy 224, 213-232.

Bowes, C., Li, T., Danciger, M., Baxter, L.C., Applebury, M.L., Farber, D.B., 1990. Retinal degeneration in the rd mouse is caused by a defect in the β subunit of rod cGMP-phosphodiesterase. Nature 347, 677.

Brazma, A., Hingamp, P., Quackenbush, J., Sherlock, G., Spellman, P., Stoeckert, C., Aach, J., Ansorge, W., Ball, C.A., Causton, H.C., 2001. Minimum information about a microarray experiment (MIAME)—toward standards for microarray data. Nature genetics 29, 365.

Costes, S.V., Daelemans, D., Cho, E.H., Dobbin, Z., Pavlakis, G., Lockett, S., 2004. Automatic and quantitative measurement of protein-protein colocalization in live cells. Biophysical journal 86, 3993-4003.

Cuenca, N., Fernandez-Sanchez, L., Campello, L., Maneu, V., De la Villa, P., Lax, P., Pinilla, I., 2014. Cellular responses following retinal injuries and therapeutic approaches for neurodegenerative diseases. Prog Retin Eye Res 43, 17-75.

De Silva, S.R., Barnard, A.R., Hughes, S., Tam, S.K.E., Martin, C., Singh, M.S., Barnea-Cramer, A.O., McClements, M.E., During, M.J., Peirson, S.N., Hankins, M.W., MacLaren, R.E., 2017. Long-term restoration of visual function in end-stage retinal degeneration using subretinal human melanopsin gene therapy. Proc Natl Acad Sci U S A 114, 11211-11216.

Dhingra, A., Tummala, S.R., Lyubarsky, A., Vardi, N., 2014. PDE9A is expressed in the inner retina and contributes to the normal shape of the photopic ERG waveform. Front Mol Neurosci 7, 60.

Du, P., Kibbe, W.A., Lin, S.M., 2008. lumi: a pipeline for processing Illumina microarray. Bioinformatics 24, 1547-1548.

Hoque, M., Rentero, C., Cairns, R., Tebar, F., Enrich, C., Grewal, T., 2014. Annexins - scaffolds modulating PKC localization and signaling. Cell Signal 26, 1213-1225.

Hughes, S., Jagannath, A., Hickey, D., Gatti, S., Wood, M., Peirson, S.N., Foster, R.G., Hankins, M.W., 2015. Using siRNA to define functional interactions between melanopsin and multiple G Protein partners. Cell Mol Life Sci 72, 165-179.

Hwang, C.K., Chaurasia, S.S., Jackson, C.R., Chan, G.C., Storm, D.R., Iuvone, P.M., 2013. Circadian rhythm of contrast sensitivity is regulated by a dopamine-neuronal PAS-domain protein 2-adenylyl cyclase 1 signaling pathway in retinal ganglion cells. J Neurosci 33, 14989-14997.

Li, Q., Lau, A., Morris, T.J., Guo, L., Fordyce, C.B., Stanley, E.F., 2004. A syntaxin 1, Gαo, and N-type calcium channel complex at a presynaptic nerve terminal: analysis by quantitative immunocolocalization. Journal of Neuroscience 24, 4070-4081.

Marc, R.E., Jones, B.W., 2003. Retinal remodeling in inherited photoreceptor degenerations. Mol Neurobiol 28, 139-147.

Nishiguchi, K.M., Carvalho, L.S., Rizzi, M., Powell, K., Holthaus, S.M., Azam, S.A., Duran, Y., Ribeiro, J., Luhmann, U.F., Bainbridge, J.W., Smith, A.J., Ali, R.R., 2015. Gene therapy restores vision in rd1 mice after removal of a confounding mutation in Gpr179. Nat Commun 6, 6006.

Pascual, I., Larrayoz, I.M., Campos, M.M., Rodriguez, I.R., 2010. Methionine sulfoxide reductase B2 is highly expressed in the retina and protects retinal pigmented epithelium cells from oxidative damage. Exp Eye Res 90, 420-428.

Peirson, S.N., 2007. Quantitative analysis of ocular gene expression, Real-time PCR. Taylor & Francis, pp. 135-154.

Pittler, S.J., Baehr, W., 1991. Identification of a nonsense mutation in the rod photoreceptor cGMP phosphodiesterase beta-subunit gene of the rd mouse. Proceedings of the National Academy of Sciences 88, 8322-8326.

Ritchie, M.E., Phipson, B., Wu, D., Hu, Y., Law, C.W., Shi, W., Smyth, G.K., 2015. limma powers differential expression analyses for RNA-sequencing and microarray studies. Nucleic acids research 43, e47-e47.

Schindelin, J., Arganda-Carreras, I., Frise, E., Kaynig, V., Longair, M., Pietzsch, T., Preibisch, S., Rueden, C., Saalfeld, S., Schmid, B., 2012. Fiji: an open-source platform for biological-image analysis. Nature methods 9, 676.

Schmitz, F., Augustin, I., Brose, N., 2001. The synaptic vesicle priming protein Munc13-1 is absent from tonically active ribbon synapses of the rat retina. Brain Research 895, 258-263.

Silvia Marino, P.K., Carly Leung, Hetty A. G. M. van der Korput, Jan Trapman, Isabelle Camenisch, Anton Berns and Sebastian Brandner, 2002. PTEN is essential for cell migration but not for fate determination and tumourigenesis in the cerebellum. Development 129, 3513-3522.

Srinivas, S., Watanabe, T., Lin, C.-S., William, C.M., Tanabe, Y., Jessell, T.M., Costantini, F., 2001. Cre reporter strains produced by targeted insertion of EYFP and ECFP into the ROSA26 locus. BMC developmental biology 1, 4.

Team, R.C., 2013. R: A language and environment for statistical computing. R Foundation for Statistical Computing, Vienna.

Vardimon, L., Fox, L.E., Moscona, A.A., 1986. Developmental regulation of glutamine synthetase and carbonic anhydrase II in neural retina. Proceedings of the National Academy of Sciences 83, 9060-9064.
