# Supplementary material for: ON-bipolar cell gene expression during retinal degeneration: Implications for optogenetic visual restoration
Source: Exp Eye Res. 2021 Jun;207:108553. doi: 10.1016/j.exer.2021.108553 (PMC8214074; doi:10.1016/j.exer.2021.108553)
Supplement: Multimedia component 2 [file mmc2.pdf]

MJ Gilhooley, D Hickey, M Lindner, T Palumaa, S Hughes, S Peirson, RE MacLaren, MW Hankins

“Retinal bipolar cell gene expression during retinal degeneration: implications for optogenetic visual restoration”

Supplementary Figures & Tables

February 2021

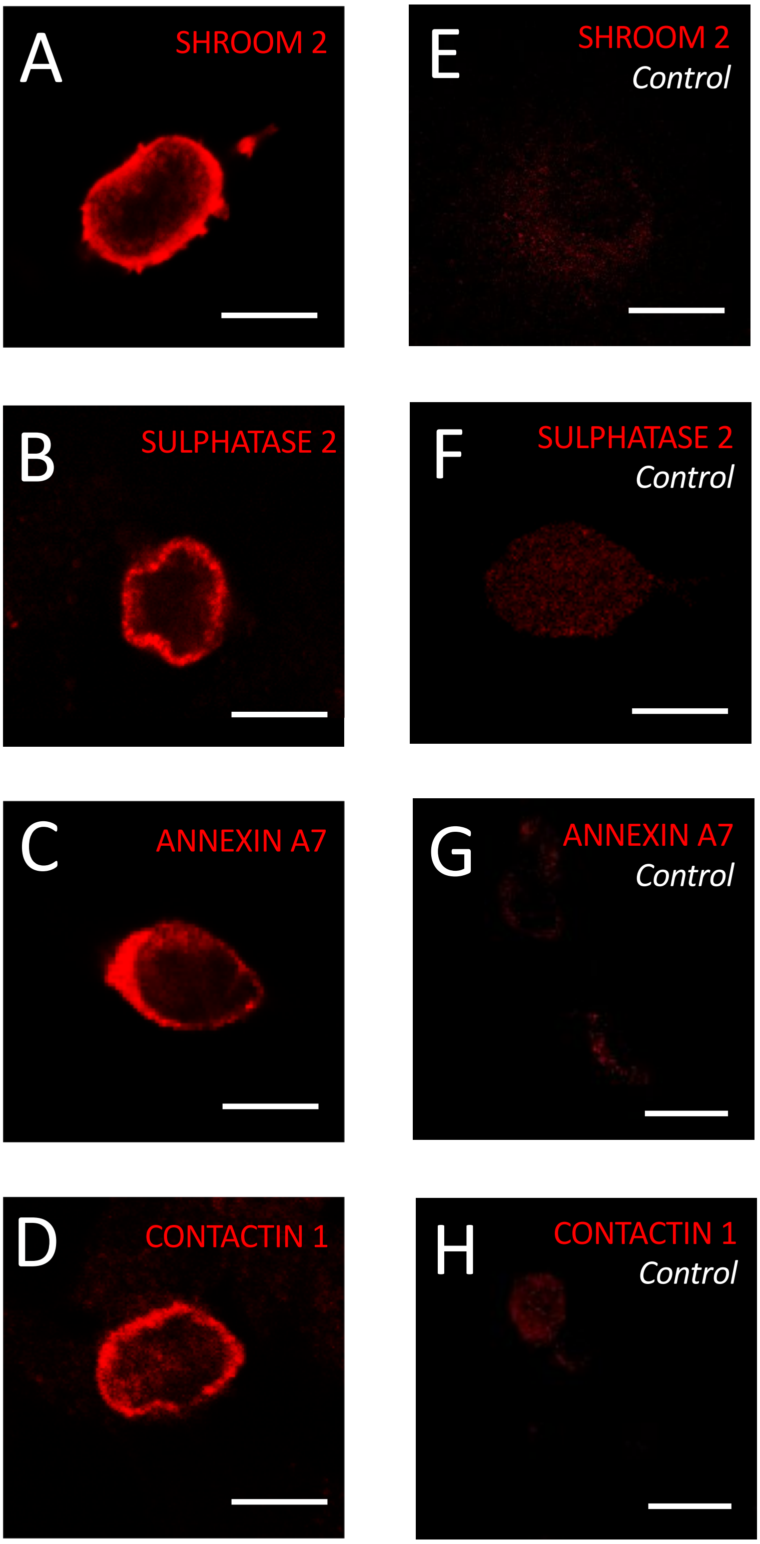
A
SHROOM 2
E
SHROOM 2
*Control*
B
SULPHATASE 2
F
SULPHATASE 2
*Control*
C
ANNEXIN A7
G
ANNEXIN A7
*Control*
D
CONTACTIN 1
H
CONTACTIN 1
*Control*

*(Fig S1.)*

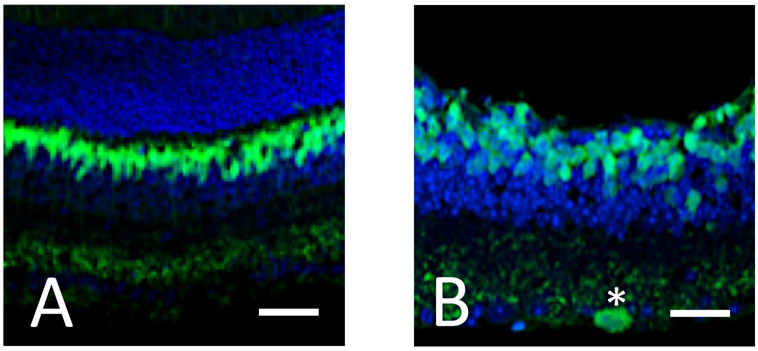
A
B
*

*(Fig S2.)*

Figure S1. Validation of antibodies. ICC images of HEK293T cells transfected to express the protein corresponding to the labelled antibody. As can be seen, all four stain at a level far above that of a non-transfected control. Note staining pattern of all four antibodies is consistent with protein in or around the cell membrane.
A – D Appropriately transfected HEK293T cells stained with the respective antibody
E – H Untransfected HEK293T cells stained with the respective antibody
Scale bars - 10μm

Figure S2. L7.Cre.EYFP mice express EYFP in rod and cone type 2 & 6 bipolar cells as well as a small subset of bistratified retinal ganglion cells (circa 1,500 in retina, denoted by asterix). Blue = DAPI, Green = YFP Scale bars = 15μm

(A) On a $Pde6b^{wt/wt}$ background
(B) On a $Pde6b^{rd1/rd1}$ background

| Probe/gene name | $log_2$ fold change | p value |
|---|---|---|
| Rod Specific | | |
| *Gnat1* | -21.14 | 0.0002 |
| *Pdc* | -1.83 | 0.0421 |
| *Pde6b* | -3.47 | 0.0004 |
| *Nrl* | -2.79 | 0.0380 |
| *Sag* | -6.01 | 0.0009 |
| *Slc24a1* | -5.27 | 0.0231 |
| *Pde6g* | -3.02 | 0.0473 |
| *Reep6* | -2.56 | 0.0380 |
| *Esrrb* | -2.00 | 0.0153 |

| Probe/gene name | $log_2$ fold change | p value |
|---|---|---|
| Rod contentious | | |
| *Gpnmb* | -4.14 | 0.0035 |
| *Ldha* | -4.61 | 0.0014 |
| *Car2* | -2.94 | 0.0004 |
| *Adcy1* | -2.97 | 0.0004 |

Table S1. (A) Genes with rod specific ontological annotation; (B) Genes with literature suggesting contentiousness due to low bipolar compared to photoreceptor protein expression. A positive log fold change represents upregulation in degenerate samples. *"p* value" represents the adjusted p-value following False Detection Rate (FDR) testing.

| Symbol | Probe/gene name | $\log_2$ fold Δ | p value |
|---|---|---|---|
| A - Probes upregulated in degeneration | | | |
| *Igfn1* | Immunoglobulin-like and fibronectin type III domain containing 1 | 4.10 | 0.0181 |
| *Cgn* | Cingulin | 3.52 | 0.0138 |
| *Msrb2* | Methionine sulfoxide reductase B2 | 3.03 | 0.0344 |
| *Cabyr* | Calcium binding tyrosine-(Y)-phosporylation regulated | 2.25 | 0.0380 |
| *Efnb1* | Ephrin-B1 | 2.15 | 0.0312 |
| *Ccdc171* | Coiled-coil domain containing 171 | 1.99 | 0.0458 |
| *Phc1* | Polyhomeotic homolog 1 | 1.93 | 0.0276 |
| *Sfxn1* | Sideroflexin 1 | 1.92 | 0.0195 |
| *Fam19a3* | Family with sequence similarity 19 (chemokine (C-C motif)-like) A3 | 1.87 | 0.0458 |
| *Shroom2* | Shroom family member 2 | 1.81 | 0.0416 |
| *Sfrs1* | Serine/arginine-rich splicing factor 1 | 1.72 | 0.0417 |
| *Sept4* | Septin 4 | 1.54 | 0.0324 |
| *Sulf2* | Sulfatase 2 | 1.43 | 0.0344 |
| B - Probes downregulated in degeneration | | | |
| *Nrm* | Nurim (nuclear envelope membrane protein) | -1.40 | 0.0416 |
| *Gm362* | Predicted gene 362 | -1.46 | 0.0138 |
| *Pde9a* | Phosphodiesterase 9A | -1.55 | 0.0416 |
| *Ndufb2* | NADH dehydrogenase (ubiquinone) 1 beta subcomplex, 2 | -1.55 | 0.0414 |
| *Gabrg2* | Gamma-aminobutyric acid (GABA) A receptor, gamma 2 | -1.56 | 0.0494 |
| *Anxa7* | Annexin A7 | -1.56 | 0.0244 |
| *Fam195a* | Family with sequence similarity 195, member A | -1.61 | 0.0053 |
| *Atox1* | Antioxidant 1 copper chaperone | -1.62 | 0.0414 |
| *Socs5* | Suppressor of cytokine signaling 5 | -1.64 | 0.0149 |
| *Csrnp2* | Cysteine-serine-rich nuclear protein 2 | -1.65 | 0.0276 |
| *Rnf11* | Ring finger protein 11 | -1.75 | 0.0244 |
| *Cntn1* | Contactin 1 | -1.79 | 0.0494 |
| *COXIII* | Mitochondrial cytochrome oxidase II subunit | -1.83 | 0.0244 |
| *Ap3m2* | Adapter-related protein complex 3, mu 2 subunit | -1.85 | 0.0231 |
| *Rnf11* | Ring finger protein 11 | -1.88 | 0.0060 |
| *Mif* | Macrophage migration inhibitory factor | -1.91 | 0.0337 |
| *Pde1c* | Phosphodiesterase 1C, calmodulin-depdendent 70kDa | -1.96 | 0.0053 |
| *Pcdha7* | Protocadherin alpha 7 | -1.98 | 0.0264 |
| *Extl3* | Exostosin-like glycosyltransferase 3 | -2.05 | 0.0291 |
| *Unc13a* | Unc-13 homolog A | -2.11 | 0.0060 |
| *Gm5478* | Predicted pseudogene 5478 | -2.13 | 0.0103 |
| *Tmsb10* | Thymosin beta 10 | -2.15 | 0.0494 |
| *Pgk1* | Phosphoglycerate kinase 1 | -2.30 | 0.0414 |
| *Stmnd1* | Stathmin domain containing 1 | -2.42 | 0.0034 |
| *Mt2* | Metallothionein 2 | -2.50 | 0.0126 |
| *Yaf2* | YY1 associated factor 2 | -2.54 | 0.0473 |
| *Clip1* | CAP-GLY domain containing linker protein 1 | -2.75 | 0.0060 |
| *Slc7a3* | Solute carrier family 7 (cationic amino acid transporter, Y+ system), 3 | -2.87 | 0.0103 |
| *Abca1* | ATP-binding cassette, sub-family A (ABC1), member 1 | -3.10 | 0.0171 |
| *Them6* | Thioesterase superfamily member 6 | -3.24 | 0.0011 |
| *Hdac9* | Histone deacetylase 9 | -3.69 | 0.0029 |
| *Ptprr* | Protein tyrosine phosphatase, receptor type, R | -3.72 | 0.0135 |
| *Naa11* | N(alpha)-acetyltransferase 11, NatA catalytic subunit | -3.83 | 0.0004 |
| *Lynx1* | Ly6/neurotoxin 1 | -8.26 | 0.0135 |
| *Mt1* | Metallothionein 1 | -10.73 | 0.0015 |

Table S2. Differentially expressed genes in a microarray study comparing FACS-isolated EYFP-positive cells from degenerate and non-degenerate L7-Cre EYFP mouse retinas. Statistically significant, differentially expressed, probes are listed according to log fold change. A positive log fold change represents upregulation in the degenerate samples. "*p* value" represents the adjusted p-value following False Detection Rate (FDR) testing.

| Probe | Log$_2$ fold Δ | p value |
|---|---|---|
| **Receptor** | | |
| *Grm6* | 1.054 | 0.8573 |
| **Gα subunits** | | |
| *Gna11* | 1.04 | 0.7896 |
| *Gna11* | -1.07 | 0.8574 |
| *Gna14* | -1.08 | 0.7764 |
| *Gnai1* | -1.09 | 0.9360 |
| *Gnai2* | -1.04 | 0.8810 |
| *Gnai3* | 1.01 | 0.9863 |
| *Gnao1* | -1.23 | 0.7900 |
| *Gnao1* | 1.02 | 0.9835 |
| *Gnaq* | -1.40 | 0.0539 |
| *Gnaq* | -1.03 | 0.8832 |
| **Gβγ subunits** | | |
| *Gnb3* | 1.91 | 0.1685 |
| *Gnb4* | -1.05 | 0.9335 |
| *Gnb5* | -1.01 | 0.9670 |
| *Gng2* | -1.10 | 0.8044 |
| *Gng3* | 1.34 | 0.8414 |
| *Gng5* | 1.04 | 0.8175 |
| *Gng7* | 1.64 | 0.6041 |
| *Gng7* | 1.07 | 0.7679 |
| *Gng10* | -1.08 | 0.7839 |
| *Gng11* | -1.05 | 0.8035 |
| *Gng13* | -1.13 | 0.7109 |
| **Bipolar cell-specific** | | |
| *Cabp5* | 1.48 | 0.5394 |
| *Pcp2* | -1.12 | 0.8044 |
| *Prkca* | -1.09 | 0.7514 |
| *Scgn* | 1.40 | 0.8044 |
| *Scgn* | -1.02 | 0.9746 |
| *Vsx1* | 1.04 | 0.9102 |
| *Vsx2* | 1.21 | 0.8056 |
| *Vsx2* | 1.10 | 0.9021 |
| **Kinases** | | |
| *Grk4* | 1.13 | 0.7241 |
| *Grk4* | -1.07 | 0.7896 |
| *Grk5* | 1.09 | 0.7918 |
| *Grk5* | 1.04 | 0.8642 |

| Probe | Log$_2$ fold Δ | p value |
|---|---|---|
| **Intracellular effectors** | | |
| *Adcy1* | -2.97 | 0.0004 |
| *Adcy1* | 1.05 | 0.8044 |
| *Adcy2* | 1.77 | 0.5721 |
| *Adcy3* | 1.01 | 0.9480 |
| *Adcy8* | 1.06 | 0.8086 |
| *Adcy9* | 1.05 | 0.8035 |
| *Pde1b* | -1.37 | 0.5764 |
| *Pde1b* | -1.41 | 0.6178 |
| *Pde1c* | -1.96 | 0.0053 |
| *Pde1c* | -1.22 | 0.1861 |
| *Pde1c* | -1.09 | 0.7629 |
| *Pde1c* | 1.19 | 0.7932 |
| *Pde1c* | -1.10 | 0.8337 |
| *Pde1c* | 1.00 | 0.9986 |
| *Pde3a* | -1.03 | 0.8542 |
| *Pde3a* | 1.01 | 0.9441 |
| *Pde3a* | 1.00 | 1.0000 |
| *Pde3b* | -1.02 | 0.9259 |
| *Pde4a* | -1.12 | 0.5721 |
| *Pde4b* | -1.26 | 0.4073 |
| *Pde4b* | -1.20 | 0.7671 |
| *Pde4b* | 1.27 | 0.8311 |
| *Pde4b* | -1.02 | 0.9409 |
| *Pde4d* | -2.05 | 0.5154 |
| *Pde4d* | -1.00 | 0.9918 |
| *Pde7a* | -1.06 | 0.7887 |
| *Pde7b* | -1.16 | 0.6154 |
| *Pde8b* | -1.02 | 0.8908 |
| *Pde9a* | -1.55 | 0.0416 |
| *Plcb3* | 1.07 | 0.7536 |
| *Plcb4* | 1.24 | 0.8044 |
| **TRP channels** | | |
| *Trpc6* | -1.05 | 0.7749 |
| *Trpc7* | -1.29 | 0.7887 |
| *Trpm1* | 1.19 | 0.7847 |
| *Trpm1* | -1.13 | 0.8035 |
| *Trpm1* | 1.09 | 0.8220 |
| *Trpm1* | 1.19 | 0.8401 |
| **Arrestin** | | |
| *Arrb2* | -1.11 | 0.8673 |

| Housekeeping genes | | | | | |
|---|---|---|---|---|---|
| *Actb* | 1.45 | 0.6114 | *Gapdh* | -1.51 | 0.1950 |
| *Actb* | -1.31 | 0.7128 | *Rplp0* | -1.15 | 0.8035 |
| *Actb* | -1.03 | 0.9801 | *Rplp0* | 1.02 | 0.9900 |
| *Actb* | -1.03 | 0.9802 | | | |

Table S3. In a microarray study comparing FACS-isolated EYFP-positive cells from degenerate and non-degenerate L7-Cre EYFP mouse retinas, genes related to intracellular communication, the native on-bipolar signal transduction cascade. A selection of "housekeeping" genes are included for comparison. A positive log fold change represents upregulation in degenerate samples. "p value" represents the adjusted p-value following False Detection Rate (FDR) testing. Note that multiple probes for some genes are reported in the above table, for clarity, all probes are labelled only with the associated gene name.

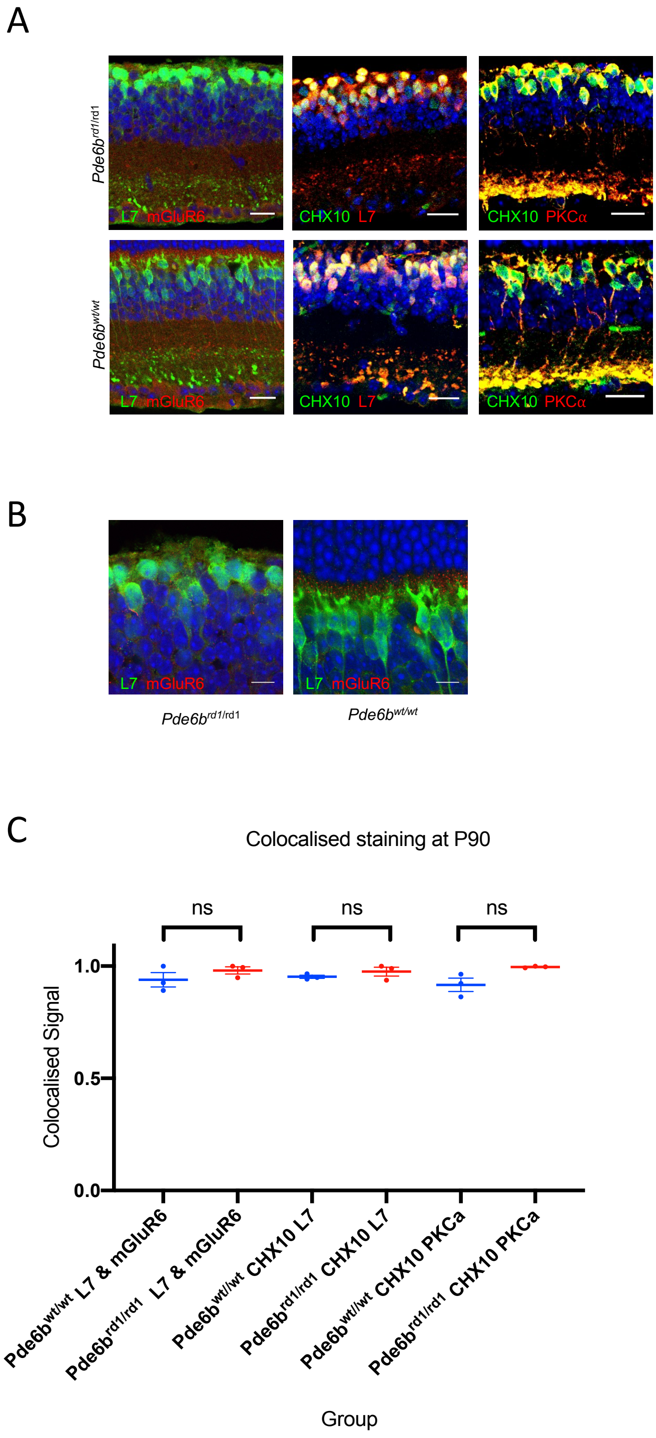
A
$Pde6b^{rd1/rd1}$
$Pde6b^{wt/wt}$
L7 mGluR6
CHX10 L7
CHX10 PKCα
B
L7 mGluR6
L7 mGluR6
$Pde6b^{rd1/rd1}$
$Pde6b^{wt/wt}$
C
Colocalised staining at P90
ns
ns
ns
Colocalised Signal
1.0
0.5
0.0
$Pde6b^{wt/wt}$ L7 & mGluR6
$Pde6b^{rd1/rd1}$ L7 & mGluR6
$Pde6b^{wt/wt}$ CHX10 L7
$Pde6b^{rd1/rd1}$ CHX10 L7
$Pde6b^{wt/wt}$ CHX10 PKCa
$Pde6b^{rd1/rd1}$ CHX10 PKCa
Group

*(Fig S3.)*

Figure S3.

A. Immunohistochemical staining of retina from *Pde6b*$^{rd1/rd1}$ and *Pde6b*$^{wt/wt}$ mice culled at P90 for proteins associated with the bipolar signalling cascade (mGluR6, PKC$\alpha$ & L7 – none of which demonstrated significant changes in gene expression between *Pde6b*$^{rd1/rd1}$ and *Pde6b*$^{wt/wt}$ - see table S3). In all panels, DAPI staining is in blue, other colours as indicated in panels scale bar = 20μm main panels.
B. Higher magnification views of mGluR6 staining in the outer plexiform layer . Note particularly the punctate pattern of staining of the bipolar cells dendrites in the wild type. Note L7 staining covers cell bodies and dendrites. Scale bars =10μm.
C. Colocalised pixels above threshold - a semiquantitative index of protein expression based on immunohistochemical staining. Normalised to highest value over all retinae stained for the same proteins. t-tests did not reveal a difference in mean values between *Pde6b*$^{rd1/rd1}$ and *Pde6b*$^{wt/wt}$ retinae stained for the same proteins ($p>0.05$). NS = not-significant.

| Probe | $Log_2$ fold Δ | p value |
|---|---|---|
| Kainate type | | |
| *Grik1* | 1.57 | 0.6786 |
| *Grik1* | 1.07 | 0.7896 |
| *Grik1* | 1.13 | 0.7934 |
| *Grik1* | 1.09 | 0.8785 |
| *Grik1* | -1.03 | 0.9705 |
| *Grik4* | -1.01 | 0.9784 |
| Vglut transporter | | |
| *Slc17a6* | -1.05 | 0.8679 |
| *Slc17a6* | -1.01 | 0.9865 |
| *Slc17a7* | -1.05 | 0.8592 |
| *Slc17a8* | -1.08 | 0.7415 |

| Probe | $Log_2$ fold Δ | p value |
|---|---|---|
| AMPA type | | |
| *Gria1* | -1.39 | 0.7587 |
| *Gria1* | 1.05 | 0.8440 |
| *Gria1* | 1.04 | 0.8681 |
| *Gria1* | 1.04 | 0.8708 |
| *Gria2* | 1.27 | 0.6526 |
| *Gria2* | 1.21 | 0.7415 |
| *Gria3* | -1.04 | 0.8035 |
| *Gria3* | -1.06 | 0.9259 |
| *Gria4* | 1.07 | 0.7587 |
| NMDA type | | |
| *Grin2c* | -1.02 | 0.9094 |

*GABA and Glycine receptor subunits*

| GABA | | |
|---|---|---|
| *Gabra1* | -1.05 | 0.8824 |
| *Gabra3* | 1.07 | 0.8194 |
| *Gabrb1* | 1.01 | 0.9632 |
| *Gabrb2* | 1.11 | 0.6142 |
| *Gabrb2* | 1.01 | 0.9870 |
| *Gabrb3* | 1.12 | 0.7500 |
| *Gabrb3* | -1.11 | 0.8013 |
| *Gabrb3* | -1.03 | 0.9382 |
| *Gabrg2* | -1.56 | 0.0494 |
| *Gabrg2* | -1.48 | 0.6302 |
| *Gabrg2* | -1.36 | 0.6308 |
| *Gabrg2* | 1.06 | 0.7420 |
| *Gabrg2* | -1.06 | 0.8721 |
| *Gabrg2* | -1.10 | 0.9477 |
| *Gabrr1* | -1.56 | 0.5706 |
| *Gabrr1* | -1.04 | 0.8718 |
| *Gabrr2* | -1.16 | 0.7896 |

| Glycine | | |
|---|---|---|
| *Glra2* | -1.06 | 0.8015 |
| *Glra4* | 1.09 | 0.7753 |
| *Glrb* | 1.07 | 0.7969 |

Table S4. In a microarray study comparing FACS-isolated EYFP-positive cells from degenerate and non-degenerate L7-Cre EYFP mouse retinas, genes relating to glutamate, glycine and gaba receptors;. A positive log fold change represents upregulation in degenerate samples. “P value” represents the adjusted p-value following False Detection Rate (FDR) testing. Note that multiple probes for some genes are reported in the above table, for clarity, all probes are labelled only with the associated gene name.

| Protein of Interest | Antibody (source & product number) | Plasmid used in validation ICC |
|---|---|---|
| **ANNEXINa7** | Abcam ab197586 | Source bioscience plasmid no. 3157926 |
| **CONTACTIN1** | Abcam ab66265 | Sino biologic plasmid no. MG50933 |
| **SHROOM2** | Atlas hpa051646 | GenScript plasmid no. OMu14281D |
| **SULPHATASE2** | Abcam ab113405 | Addgene plasmid no. 13008 |
| | | |
| **CHX10** | Abcam ab16141 | See Kim et al [REFERENCE Kim et al 2008] |
| **PKCα** | Abcam ab32376 | See Ruether et al [REFERENCE Ruether et al 2010] |

*(Table S5)*

Table S5. Antibodies and plasmids used in their validation
